# Effectiveness of Z-score of log-transformed A Body Shape Index (LBSIZ) in predicting cardiovascular disease in Korea: the Korean Genome and Epidemiology Study

**DOI:** 10.1038/s41598-018-30600-9

**Published:** 2018-08-14

**Authors:** Shinje Moon, Jung Hwan Park, Ohk-Hyun Ryu, Wankyo Chung

**Affiliations:** 1 Division of Endocrinology and Metabolism, Hallym University College of Medicine, Chuncheon, South Korea; 2 Department of Internal Medicine, Hanyang University College of Medicine, Seoul, South Korea; 3 Department of Public Health Science, Graduate School of Public Health, Seoul National University, Seoul, South Korea; 4 Institute of Health and Environment, Seoul National University, Seoul, South Korea

## Abstract

Body mass index (BMI) and waist circumference (WC) have limitations in stratifying cardio-metabolic risks. Another obesity measure, A Body Shape Index (ABSI), has been introduced but its applicability remains limited. To address this, the z-score of the log-transformed ABSI (LBSIZ) was recently developed. This study aimed to examine the ability of LBSIZ, compared to that of WC and BMI, to predict cardiovascular disease (CVD) risk. The study included 8,485 participants aged 40–69 years (mean age = 52.1) who were followed for 10 years and recruited from the Korean Genome and Epidemiology Study, a population-based cohort study. The area under the curve was 0.635 (95% confidence interval [CI]: 0.614–0.657) for LBSIZ, 0.604 (95%CI: 0.580–0.627) for WC, and 0.538 (95%CI: 0.514–0.562) for BMI. The AUC of the Framingham risk score (FRS) was 0.680 (95%CI: 0.659–0.701) in comparison. When we added LBSIZ to the model, the integrated AUC significantly improved from 0.680 to 0.692 (95%CI: 0.672–0.713; p value, 0.033), whereas there were no changes with BMI (AUC, 0.678; 95%CI: 0.656–0.699) or WC (AUC, 0.679; 95%CI: 0.658–0.701). In the multivariate Cox regression analysis, LBSIZ but not BMI or WC showed a significant hazard ratio of CVD event compared to 1st decile of each parameter. In the restricted cubic spline regression, BMI and WC showed an overall J-shaped relationship with CVD events whereas LBSIZ showed a linear relationship. LBSIZ is strongly associated with CVD risk and should predict CVD risk better than BMI and WC in the general population.

## Introduction

The World Health Organization has estimated that overweight and obesity are some of the leading global risk factors for mortality and are responsible for 4.8% of deaths worldwide^1^. In Korea, the prevalence of obesity (body mass index [BMI] ≥25 kg/m^2^) increased from 25.8% in 1998 to 31.5% in 2014, for adults aged ≥19 years, according to the Korea National Health and Nutrition Examination^2^. This change is particularly important when considering the known association between obesity and cardiovascular disease (CVD), diabetes mellitus, stroke, cancer, and death^3–6^.

To measure body fat composition accurately, computed tomography (CT), magnetic resonance imaging (MRI), dual energy x-ray absorptiometry (DXA) and PET-CT can be used^7^. Unfortunately, these methods are expensive and have limited availability and accessibility in the clinical setting. Therefore, obesity has traditionally been measured with BMI (defined as weight [kg] /height [m^2^]) because of its simplicity. However, BMI cannot discriminate between muscle and fat, or identify fat location^8^. In addition, several epidemiological studies have reported the limitations of BMI in predicting the risk of heart attack, stroke, and death^9–11^. To differentiate obesity and abdominal obesity, another obesity index, A Body Shape Index (ABSI), was recently introduced to measure waist circumference (WC) for a given weight and height^12^. ABSI is calculated by the equation:

$$[{\rm{WC}}/({{\rm{weight}}}^{2/3}\ast {{\rm{height}}}^{5/6})],$$

where the scaling exponents were estimated from a log-log regression of WC for both weight and height using data from the United States, where ABSI has been shown to be closely associated with the adult mortality. ABSI, however, has limitations in its applicability in that the scaling exponents are estimated to differ across countries and by gender. Moreover, it could have statistical issues regarding skewness and symmetry, and the respective coefficients of ABSI are inflated beyond reasonable interpretation when directly used for logistic estimation^13,14^. Subsequent research has revealed conflicting results regarding ABSI for accurate prediction of chronic diseases and mortality^15–18^.

The z-score of the log-transformed ABSI (LBSIZ) has recently been proposed to complement and improve ABSI with respect to the noted limitations. LBSIZ has been shown to predict hypertension and impaired health-related quality of life^13^. In this study, we used LBSIZ as a measure of abdominal obesity and examined its ability to predict CVD risk, compared to WC and BMI, in a Korean population.

## Results

### Baseline characteristics

A cohort of 8,485 Korean adults (4,074 men and 4,411 women) aged 40–69 years (mean age = 52.1) were analysed (Supplementary Fig. 1). Table 1 summarises the anthropometric, clinical, and biochemical characteristics of the participants according to CVD events. Among the included participants, 596 (7%) had new CVD events during the 10 years of follow-up. The incidence of CVD by follow up period is described in Supplementary Table 1. Compared with healthy participants, those with CVD events tended to be older and to have a higher BMI, WC, and LBSIZ. In addition, participants with CVD events were more likely to have higher BP, triglycerides, low-density lipoprotein cholesterol (LDL-C), fasting glucose, and HbA1c levels, lower levels of HDL-C, and a higher prevalence of hypertension and dyslipidaemia than those without CVD events. However, the two groups were not significantly different regarding other characteristics such as sex and smoking status. As for obesity measures, LBSIZ showed a positive correlation with WC (Spearman’s rho, 0.489; p value, <0.001) but a weakly negative correlation with BMI (Spearman’s rho, −0.120; p value, <0.001).

**Table 1:** Characteristics of subjects according to CVD events.

	Normal (N = 7889)	CVD events (N = 596)	p
Male sex, n (%)	3790 (48.0%)	284 (47.7%)	0.887
Age, years	51.7 ± 8.8	57.5 ± 8.1	<0.001
Smoking, n (%)			0.614
Never smoker	4583 (58.8%)	335 (56.8%)	
Ex-smoker	1204 (15.5%)	95 (16.1%)	
Current smoker	2001 (25.7%)	160 (27.1%)	
BMI, Kg/m^2^	24.5 ± 3.1	24.9 ± 3.2	0.005
Waist circumference, cm	82.5 ± 8.7	85.7 ± 8.8	<0.001
LBSIZ	−0.1 ± 1.1	0.4 ± 1.0	<0.001
Systolic BP, mmHg	120.9 ± 18.2	128.6 ± 18.6	<0.001
Diastolic BP, mmHg	80.0 ± 11.4	83.4 ± 11.3	<0.001
Fasting glucose,	86.8 ± 20.5	90.7 ± 24.1	<0.001
HBA1C. %	5.8 ± 0.9	6.1 ± 1.2	<0.001
Total cholesterol, mg/dL	190.4 ± 35.4	196.2 ± 35.2	<0.001
HDL-C, mg/dL	44.7 ± 10.0	43.3 ± 9.5	0.001
Triglycerides, mg/dL	160.8 ± 104.8	181.6 ± 101.8	<0.001
LDL-C, mg/dL	113.5 ± 33.4	116.6 ± 33.0	0.032
Diabetes Mellitus, n (%)	870 (11.1%)	137 (23.1%)	<0.001
Hypertension, n (%)	2400 (30.4%)	287 (48.2%)	<0.001
Dyslipidemia, n (%)	3740 (47.4%)	330 (55.4%)	<0.001

Abbreviations: N, number; BMI, body mass index; BP, blood pressure; HDL-C, high dense lipoprotein cholesterol, LDL-C; low dense lipoprotein cholesterol; CVD, cardiovascular diseases.

Data were presented as the means ± SD or number (%).

### Association between CVD events and obesity measures

**Figure 1:**
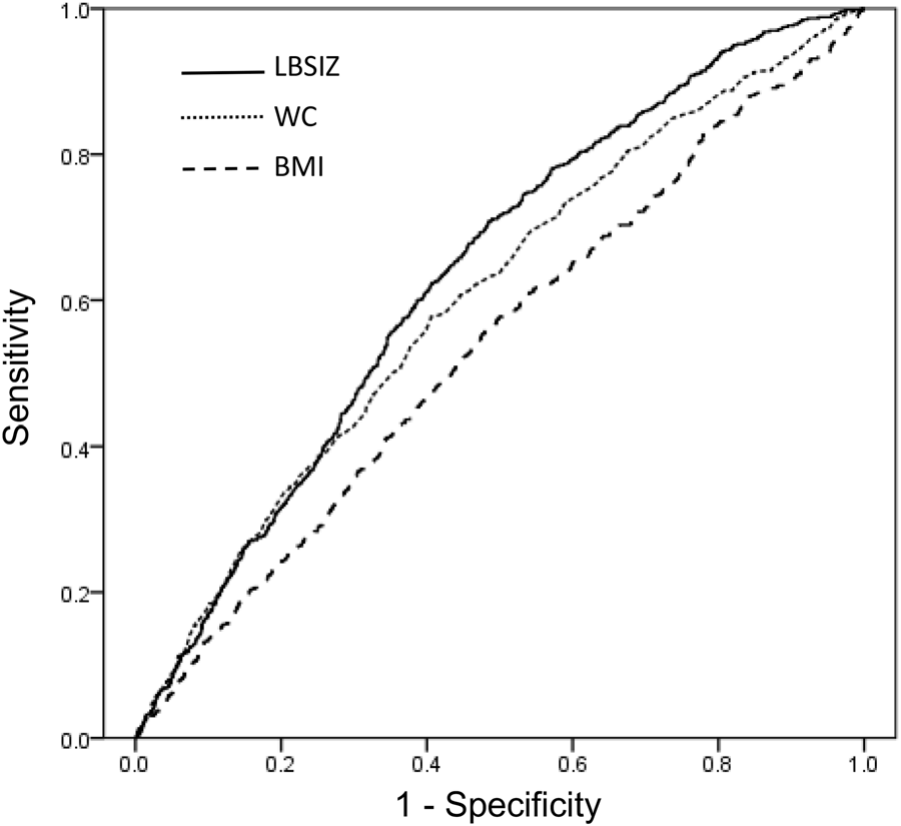
Receiver operating characteristics curves for cardiovascular events by obesity parameters.

Figure 1 shows the respective ROC curves for new CVD events during the 10 years of follow-up when using the three obesity measures. The AUC was 0.635 (95%CI: 0.614–0.657) for LBSIZ, 0.604 (95%CI: 0.580–0.627) for WC, and 0.538 (95%CI: 0.514–0.562) for BMI. The AUC of LBSIZ was significantly higher than that of WC (p value, 0.012) and BMI (p value, < 0.001). The AUC of the Framingham risk score (FRS) was 0.680 (95%CI: 0.659–0.701) in comparison. When we added LBSIZ to the model, the integrated AUC significantly improved from 0.680 to 0.692 (95%CI: 0.672–0.713; p value, 0.033), whereas there were no changes with BMI (AUC, 0.678; 95%CI: 0.656–0.699) or WC (AUC, 0.679; 95%CI: 0.658–0.701).

**Figure 2:**
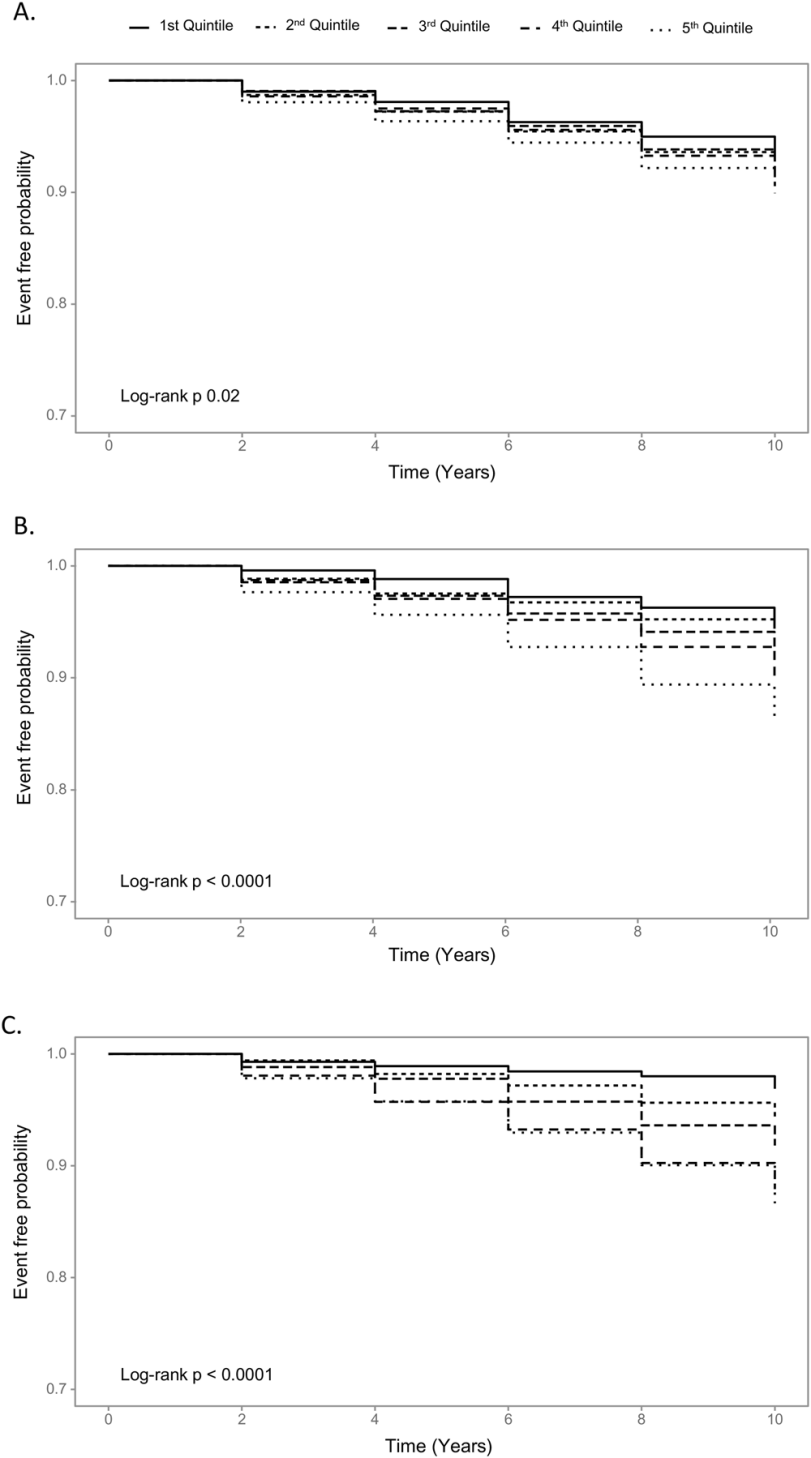
Kaplan–Meier curves of cardiovascular disease events according to the parameters for obesity. Body mass index, (**A**) Waist circumference, (**B**) LBSIZ, (**C**) LBSIZ: z-score of the log-transformed A Body Shape Index (LBSIZ).

Kaplan-Meier survival curves showed a significantly higher rate of CVD events in obese participants measured with BMI, WC, and LBSIZ compared with the non-obese ones (Fig. 2).

**Figure 3:**
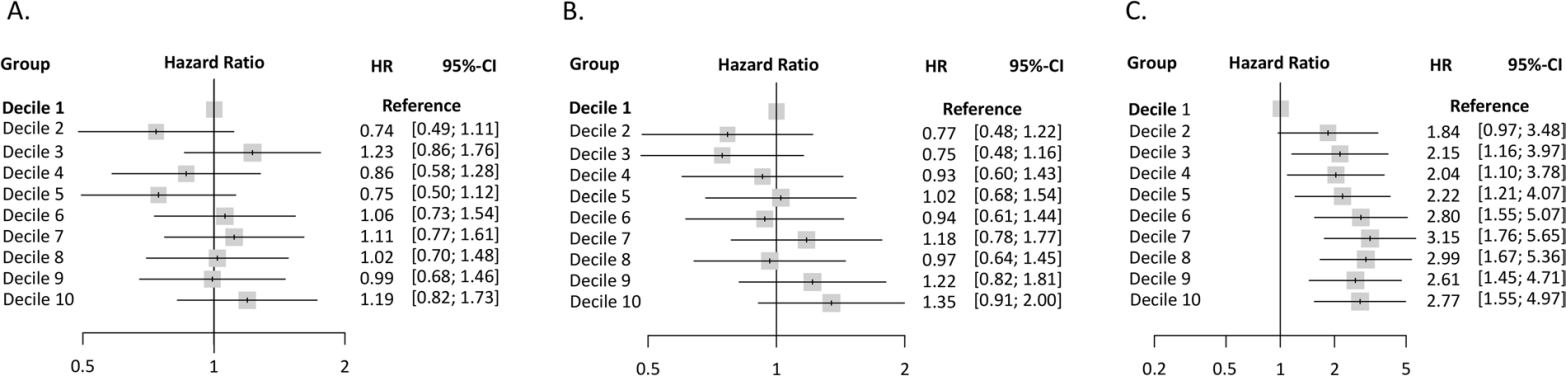
Hazard ratio (95% confidence interval) for cardiovascular events by deciles of each obesity parameter. Body mass index, (**A**) Waist circumference, (**B)** LBSIZ, (**C**) Adjusted for age, sex, smoking, systolic blood pressure, hypertension, diabetes mellitus, low-density lipoprotein cholesterol, and medication for dyslipidaemia. LBSIZ: z-score of the log-transformed A Body Shape Index (LBSIZ).

**Figure 4:**
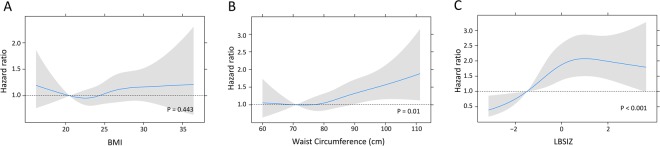
Adjusted hazard ratio of cardiovascular events by each obesity parameter. Body mass index, (**A**); Waist circumference, (**B**) LBSIZ, (**C**) Adjusted for age, sex, smoking, systolic blood pressure, hypertension, diabetes mellitus, low-density lipoprotein cholesterol, and medication for dyslipidaemia. LBSIZ: z-score of the log-transformed A Body Shape Index (LBSIZ).

We divided the three obesity measures into deciles and examined the relative risk of CVD events across the deciles using the Cox proportional hazards regression (Fig. 3). LBSIZ but not BMI or WC showed a significant HR of CVD event compared to 1^st^ decile of each parameter. In the restricted cubic spline regression, BMI and WC showed an overall J-shaped relationship with CVD events whereas LBSIZ showed a linear relationship (Fig. 4 and Supplementary Fig. 2).

## Discussion

We investigated the relationship between an abdominal obesity measure, LBSIZ, and CVD risk in a large cohort. The results of this study demonstrated that participants with higher LBSIZ values had a significantly higher rate of CVD events than those with low values and that LBSIZ is more strongly associated with CVD risk than BMI and WC in the general population. Higher BMI and WC were also associated with increased CVD risk; however, LBSIZ demonstrated a better predictive power for CVD events than BMI and WC based on the ROC curve.

Obesity, particularly the central deposition of adipose tissues, is one of the leading causes of elevated CVD mortality and morbidity^19^. Abdominal obesity has been shown to be a risk factor for CVD worldwide^20^. Several indicators of abdominal obesity such as BMI and WC are available. BMI is used to define the severity of overweight and obesity in the general population by the World Health Organization^21^. However, recent reports suggest that WC is the most practical and accurate measure of abdominal obesity for use in public health research^22,23^. Therefore, WC has replaced BMI for the clinical diagnosis of metabolic syndrome in several definitions^24^. Studies suggest that BMI and WC can reflect different types of adiposity and should not be considered as strongly correlated^25,26^. Similarly, we found that WC had a higher predictive ability for CVD than BMI.

BMI and WC have several limitations in stratifying cardio-metabolic risks. Although body fat increases and muscle mass decreases with age, changes in height, weight, and BMI may not correspond to proportional changes in body fat or muscle mass^27,28^. BMI does not consider sex differences in the distribution of fat or age-related change in muscle mass^28^. The other drawback of BMI is that it does not distinguish lean muscle from fat mass^29^. Therefore, BMI is not a specific indicator of abdominal visceral fat accumulation. A person with excessive visceral fat can have a normal BMI, ‘normal-weight obesity’, but have a high mortality risk^30^. WC is strongly associated with visceral adiposity, particularly because central obesity is a good surrogate of visceral fat accumulation and metabolic syndrome^31,32^. WC is strongly associated with metabolic risk and increased morbidity and mortality from type 2 diabetes and CVD, independent of the effect of BMI^33^. However, the drawback of WC is its inability to differentiate subcutaneous from visceral fat deposition^31^. In addition, there are insufficient normative sex- and age-specific data that would define obesity^28^.

In order to overcome these limitations of BMI and WC, many studies have tried to develop and evaluate various obesity measures. A previous study demonstrated that LBSIZ is a standard normalised obesity measure independent of weight, height, and BMI^13^. The present study also showed similar results. Therefore, LBSIZ is preferable to BMI or WC for being relatively uncorrelated with anthropometric measures. For LBSIZ to be implemented in clinical practice with less complication, we provide a supplementary template on how to calculate LBSIZ using WC, weight, and height with its related mathematical equation (Supplementary excel 1).

In this study, we demonstrated that a higher LBSIZ is associated with an increased risk of CVD. Considering the AUC of each measure, LBSIZ has a higher discriminatory capacity for CVD events compared to BMI and WC. Moreover, LBSIZ also improves the predictive power of FRS. Thus, LBSIZ can be a surrogate parameter for evaluating obesity in place of BMI and WC.

Notably, we also found that LBSIZ had a positive linear relationship with CVD events whereas BMI and WC had a J-shaped relationship. Our findings are consistent with previous studies that reported a J-shaped association of CVD events with BMI and with WC^34,35^. This J-shaped relationship of BMI and WC with CVD events has been referred to as the ‘obesity paradox’. However, results from clinical trials have unequivocally demonstrated that LDL-C, a risk factor for CVD, is dose-dependently linked to CVD risk^36^. Our findings in this study suggest that the quantification of obesity using the measurement of LBSIZ could clarify the obesity paradox.

This study has several limitations. First, we were unable to analyse fat distribution using CT or DXA dual energy x-ray absorptiometry due to lack of data. Therefore, we could not clarify how LBSIZ correlates with visceral fat, although this correlation has been shown elsewhere using another data source^13^. Second, we could not assess mortality data and may have missed fatal CVD events.

In conclusion, LBSIZ was strongly associated with the risk of CVD and can predict CVD risk better than BMI and WC in the general population. These findings may have implications in terms of CVD risk assessment in both clinical practice and epidemiologic studies. The applications of these findings are important in the context of continued increases in the prevalence of overweight and obesity.

## Materials and Methods

### Study population

This prospective cohort study collected data from two population-based cohorts from Ansan and Ansung, Korea as part of the Korean Genome and Epidemiology Study (KOGES), a Korean-government-funded survey that investigates trends in chronic non-communicable diseases. The participants were recruited from a rural area south of Seoul, Ansung, and from an industrialized area southwest of Seoul, Ansan. The participants were randomly selected and contacted via mail, telephone, or home visits. A total of 10,030 participants aged 40‒69 years were voluntarily enrolled between 2001and 2002 (Ansung, n = 5,018, response rate = 69.6%; Ansan, n = 5,012, response rate = 45.7%). A biennial follow-up examination is currently ongoing. More details regarding the design of the cohort study, the inclusion criteria, and the demographics of participants have been described elsewhere^37^.

We utilised the 10-year follow-up data for the present study. The following participants were excluded from this study: those with incomplete data (demographic, anthropometric, or laboratory), those with a clinical history of CVD or cancer at baseline, and those who had received steroids or anticoagulants. The number of eligible participants remaining was 8,485 (Supplementary Fig. 1).

### Clinical and laboratory measurements

WC was measured using a flexible tape at the narrowest point between the lowest border of the rib cage and the uppermost lateral border of the iliac crest at the end of normal expiration. Height and body weight were measured to the nearest 0.1 cm and 0.2 kg, respectively. Blood pressure (BP) was measured in the sitting position after at least 5 minutes of rest. Blood samples were obtained after an overnight fast of at least 8 hours, and biochemical assays including plasma glucose, total cholesterol, triglycerides, and high-density lipoprotein cholesterol (HDL-C) were measured using the ADVIA 1650 chemistry analyser (Bayer HealthCare Ltd., Tarrytown, NY, USA). The LDL cholesterol level was calculated using Friedewald’s equation. Haemoglobin A1c (HbA1c) level was measured using high-performance liquid chromatography (Variant II; BioRad Laboratories, Hercules, CA, USA).

The questionnaire included questions on sociodemographic information, lifestyle, personal medical history and smoking status. The participants were classified as never-, ex-, and current smokers based on their response to smoking status in the questionnaire. CVD events were investigated with a structured questionnaire. The CVD event group was defined as having ≥ 1 of the following conditions: myocardial infarction, coronary heart disease, congestive heart failure, cerebrovascular disease, or peripheral arterial disease. If the participants did not have any of the listed CVD conditions, they were classified as the normal group.

### Measurement of LBSIZ

LBSIZ was calculated by two steps: the log-transformed ABSI (LBSI) was calculated in the first step using the equation:

$$\mathrm{log}\,[{\rm{WC}}/({\exp }^{-2.69}\ast {{\rm{weight}}}^{0.73}\ast {{\rm{height}}}^{-1.06})],$$

where the scaling exponents (−2.69), (0.73) and (−1.06) were estimated from a log-log regression of WC for both weight and height using Korean data^13^. In the second step, z-score of log-transformed ABSI (LBSIZ) has been calculated using mean of LBSI (=−0.02) and standard deviation of LBSI (=0.06) (Supplementary excel 1).

### Statistical Analysis

Summary statistics are presented as mean and standard deviation with a 95% confidence interval (CI), or prevalence (%). The independent sample t-test was used to compare continuous variables and Pearson’s chi-square test was used to compare proportions according to CVD events. We analysed each measure of obesity using the receiver operating characteristic (ROC) curve for CVD to estimate the predictive ability for CVD risk. We tested if the discriminatory capacity of LBSIZ is significantly superior to that of BMI or WC. In addition, we assessed the risk of a CVD event using FRS and evaluated the integrated discriminatory capacity of FRS and each obesity parameter.

Cox proportional hazards regression models, unadjusted and adjusted, were constructed to evaluate the hazards ratio (HR) and 95% CI for CVD events. Follow-up duration was calculated as the time from the first anthropometric and clinical measures to either the date of CVD events or the end of follow-up (December 31, 2012). The graphical relationships were also evaluated with restricted cubic spline plots with 4 knots according to each obesity parameter.

Analyses were performed using the STATA/MP software (V.15.0; Stata Corporation, College Station, Texas, USA) and the statistical program R (R version 3.1.0, 2014, www.r-project.org). The significance levels were set at 0.05.

### Ethics statement

The study protocol was approved by the ethics committee of the Korean Centre for Disease Control and the institutional review board of Kangnam Sacred Heart Hospital (IRB No. HKS 2017-07-007). All participants volunteered and provided written informed consent prior to their enrolment. All participants’ records were anonymised before being accessed by the authors. All methods were carried out in accordance with the approved guidelines and regulations.

## Electronic supplementary material

Supplentary table and figures

Supplementary Excel 1
