# Supplementary material for: Effectiveness of Z-score of log-transformed A Body Shape Index (LBSIZ) in predicting cardiovascular disease in Korea: the Korean Genome and Epidemiology Study
Source: Sci Rep. 2018 Aug 14;8:12094. doi: 10.1038/s41598-018-30600-9 (PMC6092353; doi:10.1038/s41598-018-30600-9)
Supplement: Supplementary file 1 — Supplentary table and figures [file 41598_2018_30600_MOESM1_ESM.docx]

**Supplementary information for:**

**Effectiveness of Z-score of log-transformed A Body Shape Index (LBSIZ) in predicting cardiovascular disease in Korea: the Korean Genome and Epidemiology Study**

Shinje Moon^1^, Jung Hwan Park^1^, Ohk-Hyun Ryu, Wankyo Chung*

^1^ These authors contributed equally to this work as first authors.

* Corresponding author: [wankyo@snu.ac.kr](mailto:wankyo@snu.ac.kr); Phone: +82 2 880 2285; Fax: +82 2 762 2888

List of supplementary tables, figures and excel:

Supplementary table 1. Time to CVD event data

Supplementary figure 1. Flow chart for final cohort selection

Supplementary figure 2. Adjusted hazard ratio of cardiovascular events by each percentile of obesity parameter.

Supplementary Excel 1. Supplementary template to calculate LBSIZ using waist circumference, weight, and height

Supplementary table 1. Time to CVD event data

|  | Baseline | 1^st^ follow-up | 2^nd^ follow-up | 3^rd^ follow-up | 4^th^ follow-up | 5^th^ follow-up |
| --- | --- | --- | --- | --- | --- | --- |
| Normal | 8485 | 8373 | 7494 | 6799 | 6260 | 5444 |
| CVD | 0 | 112 | 108 | 124 | 132 | 120 |

Supplementary figure 1. Flow chart for final cohort selection


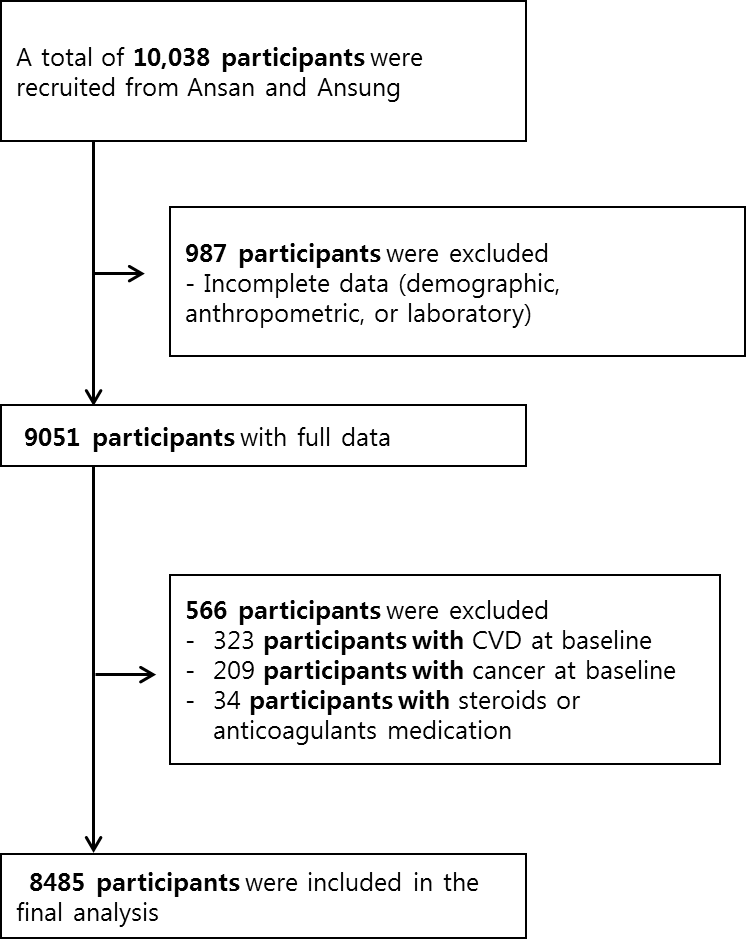


Supplementary Figure 2. Adjusted hazard ratio of cardiovascular events by each percentile of obesity parameter. Body mass index, A; Waist circumference, B; LBSIZ, C.


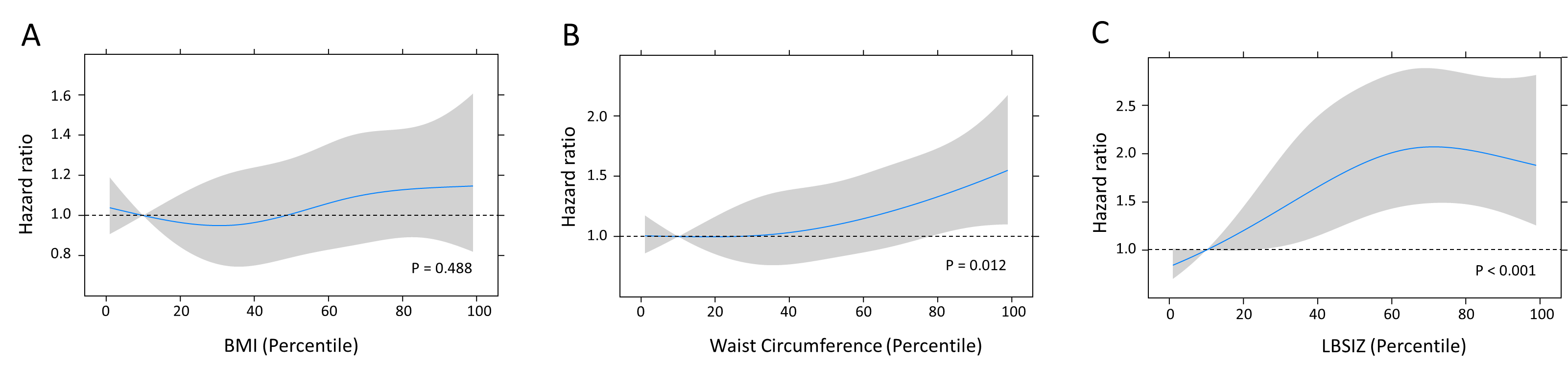


Adjusted for age, sex, smoking, systolic blood pressure, hypertension, diabetes mellitus, low-density lipoprotein cholesterol, and medication for dyslipidaemia.

LBSIZ: z-score of the log-transformed A Body Shape Index (LBSIZ)
